# Self-perceived halitosis influences social interactions

**DOI:** 10.1186/s12903-016-0189-9

**Published:** 2016-03-09

**Authors:** Ad de Jongh, Arjen J. van Wijk, Miranda Horstman, Cees de Baat

**Affiliations:** Section Social Dentistry, Academic Centre for Dentistry Amsterdam, Gustav Mahlerlaan 3004, 1081 LA Amsterdam, The Netherlands; Marketing Research Office Kien, Westerkade 15-5, 9718 Groningen, The Netherlands; Department of Oral Function and Prosthetic Dentistry, Radboud University Medical Center, PO Box 9101, Nijmegen, 6500 HB The Netherlands

**Keywords:** Halitosis, Malodor, Psychology, Psychosocial interactions

## Abstract

**Background:**

To determine the impact of self-perceived halitosis on social interactions, and the effect of using an oral rinse for management of halitosis.

**Methods:**

A survey among a representative sample of the Dutch population (*n* = 1082), and a pre-post study among a sample of consecutive coming-by volunteers (*n* = 292).

**Results:**

Participants of the representative sample rated their oral odor as 66.8 ± 17.2 and the consecutive volunteers as 70.9 ± 16.7 (range: 0–100). Sizable proportions (15.3 % and 38.1 %, respectively) indicated to always take into account their (bad) oral odor when meeting a person for the first time. The worse people perceived their oral odor, the more likely they were to take into account to keep a certain distance.

Following the use of the oral rinse, a significant decline was found of the extent to which the participants reported to take into account their oral odor when meeting a person for the first time. Both studies identified a subgroup of individuals (9.1 % and 28.1 % respectively) who reported to keep a certain distance when meeting other people, despite a “fresh” self-perceived oral odor.

**Conclusion:**

The results suggest that self-perceived oral odor negatively affects social interactions, and that adequate management of halitosis has the potential to improve such interactions.

## Background

Halitosis, often called bad breath or oral malodor, is a common condition with an estimated prevalence of 10–30 % [1, 2]. A recent research project demonstrated that halitosis is a highly unattractive aspect in social interactions. Nearly 40 % of 1006 members of an online panel reported halitosis to be the strongest “downer” when meeting a person for the first time [3]. The problem of persons with halitosis is that the halitosis may remain unnoticed because people generally are not aware of the quality of their own oral odor. Interestingly, if people are requested to judge their own oral odor, only a relatively small group of about five per cent indicates to suffer from halitosis [4]. This discrepancy between objectively assessed halitosis and self-perceived oral odor suggests a general tendency to underestimate the quality of one’s oral odor when it is bad.

One factor complicating the detection of halitosis is that people generally are reluctant or avoid to inform another person on halitosis. A study among a representative sample of the Dutch population showed that the likelihood of drawing a person’s attention to his or her halitosis decreased with the increase of the social distance to the person [3]. More specifically, it was found that while 40 % of the sample reported that he or she would draw a colleague’s attention to his or her halitosis, only less than 6 % indicated that he or she would do this to an accidentally met person.

Given the fact that it is very difficult to judge one’s own oral odor and the lack of feedback on this issue from other persons, it is conceivable that some people become uncertain about, or even fear having halitosis. This could motivate people to adjust their social activities, for example by literally taking more distance to another person, which may have a negative impact on social interactions. Conversely, it is likely that habitually using an active oral rinse for management of potential halitosis will make people less uncertain and more self-conscious, which may have positive consequences for social interactions. Although this argumentation seems logical, information on the impact of halitosis perception on the behavioral component of social interactions is lacking completely.

Therefore, two independent studies were conducted (Study A and Study B). The first aim of Study A was to derive an estimate of the self-perceived oral odor of the population of the Netherlands and to examine a number of socio-demographic correlates of self-perceived oral odor. The second aim was to test the hypothesis that the worse people perceive their own oral odor, the greater the extent to which they take into account that this person could smell one’s oral odor. The aim of Study B was to replicate the findings of Study A, and to test the hypothesis that using an active oral rinse for management of halitosis would be associated with a significant improvement of self-perceived oral odor, and to paying significantly less attention to self-perceived oral odor when meeting another person for the first time.

## Methods

Participants of Study A were members of an online survey panel assembled by the Internet survey company Panelwizard (https://www.panelwizard.com/). PanelWizard is an internationally certified panel (http://www.iso.org/iso/home/standards.htm) and comprises a total of more than 30,000 active individuals aged 16 years and older, being representative for the population of the Netherlands with regard to gender, age, marital status, education level, and employment status. The panel uses a calibration method for national and regional samples which is annually updated with the latest figures of the Dutch population. This is done in collaboration with the Dutch Central Bureau of Statistics (CBS) [5].

For the purpose of Study A an independent, random sample of 1586 panel members was invited to participate. Of them, 1083 (68 %) agreed to participate within a previously determined time frame of four days.

For Study B, potential participants were personally approached at a square in the entertainment area of the city Haarlem (152,000 inhabitants) in The Netherlands. At two consecutive Saturday evenings, two employees promoting an oral rinse to manage halitosis interviewed consecutive coming-by volunteers aged 16 years and older. Of the 500 people approached, 292 agreed to participate and provided written informed consent after explanation of the study, of whom 58.3 % were women, whereas the gender of two participants was not registered.

The survey of Study A contained two questions: ‘Some people have a fresh oral odor and other people do not. In general, how do you judge your oral odor?’ and ‘When you meet somebody for the first time, how frequently do you take into account that this person could smell your oral odor, for example by adapting a certain distance to the person?". The responses to the first question indicated their self-perceived oral odor on a visual analogue scale ranging from 0 (”extremely bad”) to 100 (”very fresh”). The second question pertained to the social functioning of the participants with the following response options: “never”, “almost never”, “sometimes”, “often”, or “always”. With regard to marital status, a distinction was made between single and cohabiting/married with/without children. Education level, defined as the highest level of education completed, was categorized into “low” (no education, only primary education, continuation/lower vocational education, lower general secondary education, or first 3 years of higher general secondary education or pre-university education), “middle” (middle vocational education, lower general secondary education, pre-university education and first year of university) and “high” (higher vocational education, bachelor or master degree). Employment status was categorized as: “unemployed”, “full-time employed”, “part-time employed”.

The participants of Study B answered five questions about gender, age, marital status, education level, and employment status. Further, they received a test sample of the promoted oral rinse (consisting of the following ingredients: Aqua, Glycerin, Hydrogenated Starch Hydrolysate, Alcohol, Zinc Acetate Dihydrate, Chlorhexidine Diacetate, Sodium Fluoride, PEG-40 Hydrogenated Castor Oil, Potassium Acesulfame, Citric Acid, Aroma). They were asked to rinse their mouth with the test fluid, every day in the morning, and to do that for one minute, using the cap (with a capacity of 10 ml.) of the bottle. The participants were requested to provide their email addresses, which were received from 213 (73 %) of them. Four days later, they received an email with a direct link to the survey consisting of four questions: ‘Did you use the test sample during a few days?’ (“yes” or “no”), How satisfied are you with how the product contributes to improving your oral odor?’ (“very satisfied”, “satisfied”, or “dissatisfied”). The other two questions were the same as used in Study A. Fifty-seven participants (27 %) completed the questionnaire, of whom 66.1 % were women, whereas the gender of one participant was not registered.

### Ethics, consent and permissions

Both studies were conducted by research agency Kien, The Netherlands, in accordance with ISO 20252 (market research) and ISO 26362 (access panels) standards, and performed in accordance with the precepts and regulations for research as stated in the Declaration of Helsinki, and the Dutch Medical Research on Humans Act (WMO) concerning scientific research. The WMO was not applicable to the present study because (a) the two surveys contained only a small number of items, (b) the study lacked random allocation (c) no `physical infringement of the physical and/or psychological integrity of the individual’ was to be expected. Written informed consent was obtained from each participant of the study. The data collected, were obtained directly by the survey company, which prepared and delivered a de-identified SPSS data file to the first author.

### Statistical analysis

The survey data were analyzed using the computer program Statistical Package for Social Sciences (SPSS) 19.0. Statistical tests included the Student’s *t*-test, the Mann–Whitney *U* test for comparing ordinal measurements, the paired-samples *t*-test, the Wilcoxon signed-rank test for ordinal data, and analysis of variance (ANOVA) for comparisons of continuous variables. Chi-square tests were used for associations between categorical variables. To test whether the distribution of socio-demographic variables of the sample was similar to the general population aged 16 years and older, a chi-square test was used to compare the observed study frequencies with the expected frequencies based on population data. To be able to interpret the participants’ judgments of self-perceived oral odor, a score ≤ 30 was considered “bad” oral odor, and a score ≥ 70 “fresh” oral odor. Multiple logistic regression analysis was used to examine the unique relationship between self-perceived oral odor and the extent of taking into account one’s oral odor, while controlling for all other factors in the model, being gender, age, marital status, education level and employment status. For this purpose the variable”taking into account one’s oral odor” was rescored into 1 (”never” to “almost never”) and 2 (”sometimes” to “always”). All demographic variables were entered into the equation, for self-perceived oral odor the forward LR selection method was used. The strength of the association between self-perceived oral odor and the extent of taking into account one’s oral odor was determined using the odds ratio and 95 % confidence intervals.

For the purpose of Study B, a power analysis was performed to determine the number of subjects needed to detect a medium effect size (es = 0.5). Using a paired-samples *t*-test, to compare the mean self-perceived oral odor before and after using the mouth rinse, es = 0.5, alpha = 0.05 and a power of 80 %, results in a required sample size of at least 34 [6]. Paired t-tests were used to compare participants’ pre- and post-test scores on self-perceived oral odor. A *P-*value of less than 0.05 was considered to indicate statistical significance.

## Results

### Socio-demographic correlates of self-perceived oral odor (Study A)

The socio-demographic characteristics of the participants of Study A are presented in Table 1. The distribution of these characteristics proved to be an accurate reflection of the 2012 data of the population of the Netherlands aged 16 years or older, in terms of gender, age, marital status, education level, and employment status (5).

**Table 1:** Demographics of the two samples, and a comparison of demographic data of study samples A and B with the general population of the Netherlands aged 16 years and older

Variable	Study A (*n* = 1082)		Outcome chi-square tests study sample A vs. Dutch population	Study B (*n* = 292)		Outcome chi-square tests study sample A vs. Dutch population	% in Dutch population
	*n*	%		*n*	%		
Gender			(1) = 1.45, *p* = 0.23			(1) = 3.38, *p* = 0.07	
Women	522	48.2		169	58.3		50.8
Men	560	51.8		121	41.7		49.2
Age			(4) = 2.49, *p* = 0.65			(4) = 13.3, *p* = 0.01	
16–29	219	20.2		86	29.5		18.2
30–39	197	18.2		40	13.7		17.9
40–49	194	17.9		40	13.7		20.1
50–59	180	16.6		52	17.8		16.8
≥ 60	292	27.1		74	25.3		27.0
Marital status^a^			(2) = 0.05, *p* = 0.98			(2) = 2.29, *p* = 0.32	
Single	217	20.1		72	24.8		20.1
Cohabiting/married without Children	509	47.1		120	41.4		46.6
Cohabiting/married (with children)	356	32.9		98	33.8		33.3
Education level^a^			(2) = 0.55, *p* = 0.76			(2) = 24.8, *p* < 0.001	
Low	393	36.3		69	23.7		37.4
Middle	410	37.9		96	33.0		38.1
High	279	25.8		126	43.3		24.5
Employment status			(2) = 0.94, *p* = 0.62			(2) = 5.38, *p* = 0.07	
Full-time	383	35.4		96	32.9		34.7
Part-time	212	19.6		86	29.5		21.3
Unemployed	487	45.0		110	37.7		44.0

^a^data of two participants are missing, all analyses were performed using the Chi^2^-test

Of the 1082 participants, 522 were women (48.2 %). The education level was significantly lower in women than in men (Z = − 4.45; *P* < 0.001). Women worked less full-time, worked more part-time and were more unemployed than men (chi-square test (2) = 264.4; *P* < 0.001).

On average, the participants judged their oral odor by a score of 66.8 ± 17.2; 4.2 % judged their oral odor “bad” (score ≤ 30), whereas more than half (56.3 %) judged their oral odor “fresh” (score ≥ 70).

A statistically significant difference in self-perceived oral odor was found among the age groups [F (4, 1077) = 7.8; *P* < 0.001]. Post-hoc comparisons showed that the participants aged 60 years and older judged their oral odor statistically significantly more fresh (M = 71.5 ± 16.4) than the people of the four younger age groups (M = 65.1 ± 17.4). Furthermore, marital status was found to be statistically significantly associated with self-perceived oral odor; participants who were cohabiting or married having children had the lowest scores on self-perceived oral odor [F (3, 1078) = 2.94, *P* = 0.03].

### The association between self-perceived oral odor and the extent of taking into account ones oral odor (Study A)

Approximately half of the participants (50.2 %) of Study A indicated that they, when meeting a person for the first time (“sometimes” or “often”), took into account that the other person could smell his or her oral odor, for example by keeping a certain distance. About 15 % reported to be “always” aware of their oral odor. Women reported significantly more often than men (19 % vs. 12 %) to “always” take into account their oral odor [chi-square test(4) = 10.2; *P* = 0.037].

The extent to which self-perceived oral odor was taken into account was also dependent on age. That is, participants younger than 60 years of age took into account this more often (“sometimes” to “always”) than participants aged 60 years and older [(69.9 % vs. 53.8 %) chi-square test(4) = 29.3; *P* < 0.01]. Further, more participants with a low education level took into account their oral odor “always” when meeting a person for the first time than participants with a high education level (18 % vs. 14 %) [chi-square test(8) = 18.4; *P* < 0.01].

There was a statistically significant difference in self-perceived oral odor among participants who “never”, “almost never”, “sometimes”, “frequently”, and “always” took into account that another person might be able to smell their oral odor (ANOVA; *P* < 0.001). In short, the worse people perceived their oral odor, the more they took into account to keep a certain distance when meeting a person for the first time (Table 2). A remarkable exception of this pattern was the group of participants who indicated that they took into account their oral odor “always”, as these 166 participants (15.3 %) judged their oral odor “fresh”. A frequency analysis of self-perceived oral odor within this group showed that 59 % (9.1 % of all participants; 60.2 % women) judged their oral odor “fresh”.

**Table 2:** Self-perceived oral odor (0–100) (mean ± s.d.) in five groups in relation to the extent to which participants of Study A indicated to take into account their oral odor, with statistically significant differences between groups indicated (*P* < 0.05)

Taking into account	Number	*Mean ± s.d.	Differences
1. “Never”	155	73.8 ± 15.8	2, 3, 4, 5
2. “Almost never”	218	69.6 ± 15.4	1, 3, 4
3. “Sometimes”	305	64.3 ± 15.5	1, 2
4. “Often”	238	62.5 ± 18.6	1, 2, 5
5. “Always”	166	67.3 ± 19.2	1, 4
Total	1082	66.8 ± 17.2	

*p < 0.05: Mean scores compared using a one-way ANOVA, between group differences based on post hoc tests (LSD)

A statistically significant association was found between gender and taking into account one’s oral odor [chi-square test(4) = 10.3; *P* = 0.04]. This association is due to the overrepresentation of women in the group who indicated that they always took into account their oral odor when meeting a person for the first time.

The results of the logistic regression analysis showed that the model was significant, chi-square test(12) = 87.1, *p* < 0.001, and adequately fitted the data, chi-square test(8) = 9.97, *p* = 0.27 (Hosmer-Lemeshow Goodness of Fit test). Self-perceived oral odor was a significant predictor for taking into account one’s oral odor (odds ratio = 0.98; 95 %: CI 0.97–0.99). In summary, the worse their self-perceived oral odor was, the more they would take into account their breath. Other significant variables in the model were age and marital status. Older people less often took their oral odor into account than younger people, and participants living in a household with children less often than participants who were single.

### Socio-demographic correlates of self-perceived oral odor (Study B)

In Table 1 the socio-demographic characteristics of the participants of Study B are presented. The distribution of these characteristics differed significantly from the 2012 data of the population of the Netherlands aged 16 years or older, in terms of age and education level (5).

The participants judged their oral odor on average 70.9 ± 16.7. The proportion of participants rating their oral odor “bad” (score ≤ 30) was 3.7 %, while the proportion of those judging it “fresh” (score ≥ 70) was 60.9 %.

### The association between self-perceived oral odor and the extent of taking into account ones oral odor (Study B)

Approximately one third of the participants (33.2 %) indicated that they, when meeting a person for the first time, took into account “sometimes” or “often” that the other person could smell his or her oral odor, for example by keeping a certain distance. The proportion of participants who reported to take into account this “always” was 38.1 %.

Table 3 displays participants’ self-perceived oral odor before and after the use of the oral rinse in relation to the extent to which they reported taking into account one’s oral odor. A significant difference was found among the scores of self-perceived oral odor of the groups differing with respect to the extent to which they reported to take into account their oral odor when meeting a person for the first time [F (4, 286) = 2,78; *P* = 0.03]. Post hoc analyses revealed that participants who indicated taking into account their oral odor “sometimes”, rated the quality of their oral odor significantly worse than those who indicated to do this “always” or “never”.

**Table 3:** Self-perceived oral odor (mean ± sd) of participants of Study B before and after the use of the oral rinse in relation to the extent to which the participants reported taking into account one’s oral odor

	Before using the oral rinse*		After using the oral rinse	
	Mean ± sd	*n* (%)***	Mean ± sd	*n* (%)***
“Never”	73.7 ± 16.3	59 (20.3)	85.0 ± 7.1	10 (20.0)
“Almost never”	71.2 ± 9.0	24 (8.2)	75.0 ± 10.7	8 (16.0)
“Sometimes”	64.9 ± 17.6	55 (18.9)	77.1 ± 9.8	17 (34.0)
“Often”	69.0 ± 12.1	42 (14.4)	82.0 ± 14.8	10 (20.0)
“Always”	72.9 ± 18.6	111 (38.1)	64.0 ± 20.7	5 (10.0)
Total	70.8 ± 16.7**	291	78.0 ± 12.9**	50

*p < 0.05: mean scores before the use of oral rinse were compared using a one-way ANOVA, between group differences based on post hoc tests (LSD). **p < 0.05: Total pre- and posttest (oral odor) scores were compared using a paired *t*-test. ***p < 0.05: Differences in the extent to which participants take into account their oral odor, before and after oral rinse, using the Wilcoxon signed rank test

### The impact of an active oral rinse on people’s self-perceived oral odor, and on taking into account one’s oral odor (Study B)

A total of 57 participants (19.5 % of the 292 people who received the oral rinse) completed the online questionnaire after having used the oral rinse during four days. Fifty of them reported having actually used the oral rinse. A paired *t*-test showed a significant improvement of self-perceived oral odor among the participants who reported having used the oral rinse (16 men and 33 women: 1 missing gender); [t(49) = 4.41; *P* < 0.001; mean difference = 11.0 ± 17.6; *d* = 0.63].

Following the use of the oral rinse, a significant decline was found of the extent to which the participants reported to take into account their oral odor (Z = −2.65; *P* = 0.008). Ninety-eight per cent of the participants who indicated that they had used the oral rinse reported to be “satisfied” or “very satisfied”. Those who indicated to be”very satisfied” reported a stronger improvement (M = 21.5 ± 20.4) in self-perceived oral odor than those who were”satisfied” [M = 8.33 ± 14.04; t (47) = −2.57; *P* = 0.013].

Although the ANOVA test was not significant, post hoc analyses showed that the group reporting to take into account their oral odor “always” when meeting a person for the first time, scored significantly lower on self-perceived oral odor than the groups reporting “never”, “almost never”, “sometimes”, and “frequently”.

Of the group of 111 participants (38.1 % of the total group) who reported to take into account their oral odor “always” when meeting a person for the first time, 82 (73.9 % being 28.1 % of the total sample; 46 women and 36 men) judged their oral odor”fresh” (≥70). After having used the oral rinse this percentage dropped to 10, 5 participants of the 50 who had used the oral rinse.

## Discussion

Only a small proportion of participants judged their oral odor as ”bad”. In both studies, the percentage of participants rating their oral odor”bad” was relatively low, 4.2 and 3.7 respectively. This finding is close to the 5 % found in one of the few studies that collected data of self-perceived oral odor [4]. The results were supportive of the presumption that people who believe that they suffer from halitosis are more likely to keep a certain distance to a person when meeting this person for the first time than people who believe that they do not suffer from halitosis.

About 15 % of the participants of Study A stated that they took into account their oral odor “always”, women and participants younger than 60 years of age significantly more frequently than men and participants older than 60 years of age. The results of Study B revealed a much larger proportion (38.1 %) of participants who indicated taking into account their oral odor “always”. This difference may be explained by differences in sample characteristics and the fact that the participants of Study B were a select population, actually consecutive coming-by persons in an entertainment area and people of this group who were prepared to participate in a study on the use of an active oral rinse for management of halitosis. Perhaps this population was more likely to be sensitive for halitosis and cautious with respect to their oral odor.

The results of Study B also showed that following the use of an active oral rinse, the participants not only reported an improved oral odor, but also noted a significant decline in the reported extent to which they took into account their oral odor. The percentage of participants, who indicated taking into account their oral odor “always” when meeting a person for the first time, dropped statistically significantly from 38 to 10 after having used the oral rinse. Thus, the preventive use of the rinse seemed rather effective in influencing a person’s behavior. These effects could be interpreted as particular important for individuals who feel insecure about their oral odor in social interactions.

Although a behavioral pattern of keeping a certain distance when having halitosis is not noteworthy intrinsically, Study A detected a subgroup of participants who reported to keep a certain distance when meeting a person for the first time, despite a “fresh” self-perceived oral odor. This counter-intuitive behavioral response may be a manifestation of social insecurity and thus a symptom of social anxiety, or a fear of rejection [7], which, in this study, was probably due to the negative effects of halitosis. The relationship between social anxiety and halitosis has been investigated previously [8]. The same holds true for the relationship of social anxiety with halitophobia, or delusional halitosis, an extremely overstated concern about having halitosis [9] in that social anxiety, avoidance behavior, and social isolation are common outcomes of halitophobia [7].

One limitation of the fact that halitosis was not objectively assessed was that it was not possible to determine which participants judged their oral odor correctly, incorrectly, or responded socially desirable. However, objective measurement of oral odor (i.e., sulfides) was not the aim of the present study, because people are not affected emotionally by the *objective* quality of their odor, but rather by their *perceptions*, and thoughts about it, and it is this that determines their behavioral response. Further, the fact that they responded anonymously online, probably made them feeling relatively free to report honestly about their perceptions. A limitation of Study B is the small sample size and a rather low response rate which means that we should be cautious when interpreting the results. Another limitation is that the effects of the oral rinse were only measured before and after using it. We did not employ a control group and the relative effects of other interventions could not be examined. For example, because the oral rinse was not tested against a placebo oral rinse it remains unclear whether the effects in Study B were due to the use of the oral rinse or, for instance, participants’ belief being able to exert some sort of control over their oral odor. Although it is impossible to tease out the effects of the rinse and something unknown that happened coincident in time with the intervention, the result that the participants reported a change regarding their social behavior is promising. Future research should conduct a longer follow-up to establish whether the positive effects associated with such interventions last in the long term.

## Conclusions

The results of both studies demonstrate that self-perceived oral odor is a potential source of social insecurity, suggesting that a negative self-perceived oral odor or halitosis-induced social fear influences social interactions and, consequently, creates distance between people literally. The study results further suggest that using an active oral rinse for management of halitosis, may lead to a significant improvement of self-perceived oral odor and to paying significantly less attention to self-perceived oral odor when meeting another person for the first time. This may enable people to become more self-confident, which may render long-lasting positive emotional and behavioral effects.

## Abbreviations

ANOVA: analysis of variance
ISO: International Organisation for Standardization
SPSS: Statistical Package for Social Sciences
WMO: Wet Medisch Wetenschappelijk Onderzoek (Dutch Medical Research on Humans Act)
